# Acetaminophen induces JNK/p38 signaling and activates the caspase-9-3-dependent cell death pathway in human mesenchymal stem cells

**DOI:** 10.3892/ijmm.2015.2254

**Published:** 2015-06-19

**Authors:** GIOU-TENG YIANG, YUNG-LUNG YU, KO-TING LIN, JEN-NI CHEN, WEI-JUNG CHANG, CHYOU-WEI WEI

**Affiliations:** 1 Department of Emergency Medicine, Taipei Tzu Chi Hospital, Buddhist Tzu Chi Medical Foundation, New Taipei 231, Taiwan, R.O.C.; 2 Department of Emergency Medicine, School of Medicine, Tzu Chi University, Hualien 970, Taiwan, R.O.C.; 3 Graduate Institute of Cancer Biology and Center for Molecular Medicine, China Medical University, Taichung 404, Taiwan, R.O.C.; 4 Department of Biotechnology, Asia University, Taichung 413, Taiwan, R.O.C.; 5 Department of Nutrition, Master Program of Biomedical Nutrition, Hungkuang University, Shalu, Taichung 433, Taiwan, R.O.C.; 6 Department of Nursing, Hungkuang University, Shalu, Taichung 433, Taiwan, R.O.C.

**Keywords:** acetaminophen, c-Jun N-terminal kinase, caspase, human mesenchymal stem cells

## Abstract

Acetaminophen (APAP) is a widely used analgesic and antipyretic drug. Generally, the therapeutic dose of APAP is clinically safe, however, high doses of APAP can cause acute liver and kidney injury. Therefore, the majority of previous studies have focussed on elucidating the mechanisms of APAP-induced hepatotoxicity and nephrotoxicity, in addition to examining ways to treat these conditions in clinical cases. However, few studies have reported APAP-induced intoxication in human stem cells. Stem cells are important in cell proliferation, differentiation and repair during human development, particularly during fetal and child development. At present, whether APAP causes cytotoxic effects in human stem cells remains to be elucidated, therefore, the present study aimed to investigate the cellular effects of APAP treatment in human stem cells. The results of the present study revealed that high-dose APAP induced more marked cytotoxic effects in human mesenchymal stem cells (hMSCs) than in renal tubular cells. In addition, increased levels of hydrogen peroxide (H_2_O_2_), phosphorylation of c-Jun N-terminal kinase and p38, and activation of caspase-9/-3 cascade were observed in the APAP-treated hMSCs. By contrast, antioxidants, including vitamin C reduced APAP-induced augmentations in H_2_O_2_ levels, but did not inhibit the APAP-induced cytotoxic effects in the hMSCs. These results suggested that high doses of APAP may cause serious damage towards hMSCs.

## Introduction

Acetaminophen (APAP) is commonly used as an analgesic and antipyretic agent (1–3), and is considered safe at therapeutic doses (4). It is readily available, and high doses of APAP may be provided to patients over a short time-period. However, APAP is the most common drug to cause clinical hepatotoxicity and nephrotoxicity in several countries (5–7). A number of studies have demonstrated that high-dose APAP (10–15 g) causes serious damage to liver and renal cells (8,9). High-dose APAP can increase the levels of reactive oxygen species (ROS), thus increasing cellular oxidative stress and causing liver and renal injury (10–12). Therefore, several studies have examined the ability of antioxidants to target high-dose APAP-induced liver and renal damage through the reduction of cellular ROS levels and oxidative stress (13–16). At present, N-acetylcysteine (NAC), an antioxidant, has been used to treat APAP-induced hepatotoxicity and nephrotoxicity in emergency cases (17–19).

In order to improve the understanding of the mechanisms underlying APAP-induced toxicity, several animal and cell models have been developed for hepatotoxic and nephrotoxic investigations. In general, high-dose APAP (>5 mM) is used to induce cell death in renal and liver cell models (20–26), and high-dose APAP (300–2,500 mg/kg) is used to induce liver and kidney damage in animal models (27–31). These studies have observed that APAP can stimulate apoptotic or necrotic death pathway activation in different cell models (24,31,32). In addition, several cellular effects and signals are stimulated in high-dose APAP-treated cells, including increased levels of ROS and oxidative stress, decreased levels of glutathione, induction of the mitogen-activated protein kinase (MAPK) signaling pathway and activation of caspase cascades (21,25,26,31,33–36).

High-dose APAP-induced clinical intoxication is predominantly found in liver and renal cells; therefore, the majority of previous studies have focussed on the mechanisms underlying high-dose APAP-triggered liver and renal injury (17,37,38). Furthermore, certain studies have indicated that APAP can exhibit antitumor activities in certain tumor types, including breast cancer, liver cancer and neuroblastoma (26,39–43). These studies also demonstrated that APAP-induced cell death is linked to nuclear factor-κB, the B-cell lymphoma 2 family or glycogen synthase kinase-3 in different tumor cells.

At present, with the exception of liver, renal and tumor cells, almost no cellular effects have been reported in other human cells following APAP therapy (10,12,39–43). Therefore, whether APAP causes toxic cellular effects in other human cells remains to be elucidated. APAP can freely cross the placenta (44,45); thus, high-dose APAP can cause cellular damage in maternal as well as fetal liver cells. In addition, several previous studies have suggested that stem cells are critical during fetal development (46–48). However, whether APAP can induce toxic cellular effects in stem cells during fetal development remains to be elucidated. APAP-induced cellular effects in human stem cells have not been reported previously, therefore, the aim of the present study was to investigate the cellular responses of APAP-treated human stem cells.

Based on the above-mentioned studies, the aim of our study was to determine the cytotoxic effects of APAP on human mesenchymal stem cells (hMSCs). Furthermore, the ROS levels (H_2_O_2_ and O_2_^−^) and the role of caspase death pathways and MAPK signaling pathways were also determined in the APAP-treated hMSCs.

## Materials and methods

### Chemicals

Caspase-3, caspase-8, caspase-9, cleaved caspase-3, cleaved caspase-8 and cleaved caspase-9 monoclonal antibodies were purchased from Cell Signaling Technology, Inc. (Danvers, MA, USA). Extracellular-signal-regulated kinase (ERK), p38, JNK, phosphorylated (p)-p38, p-ERK and p-JNK monoclonal antibodies were purchased from BD Transduction Laboratories (San Diego, CA, USA). Secondary mouse anti-human antibody was purchased from GE Healthcare (Piscataway, NJ, USA). Tubulin monoclonal antibody, luminol, lucigenin, vitamin C and Hoechst 33342 were purchased from Sigma-Aldrich (St. Louis, MO, USA). The 3-(4,5-dimethylthiazol-2-yl)-2,5-di-phenyltetrazolium bromide (MTT) kits were purchased from Bio Basic, Inc. (Markham, ON, Canada). Fetal bovine serum, Dulbecco’s modified Eagle’s medium (DMEM), DMEM-low glucose (DMEM-LG), non-essential amino acid L-glutamine and penicillin/streptomycin were obtained from GE Healthcare Life Sciences (Logan, UT, USA).

### Cells and cell cultures

The NRK-52E rat renal tubular cells were obtained from Bioresource Collection and Research Center (Hsinchu, Taiwan). The hMSCs (Bioresources Collection and Research Center, Hsin Chu, Taiwan) were cultured in DMEM-LG supplemented with 10% fetal bovine serum, 2 mM L-glutamine, 100 IU/ml penicillin/streptomycin and 0.1 mM non-essential amino acids. The NRK-52E cells were cultured in DMEM supplemented with 10% fetal bovine serum, 2 mM L-glutamine, 100 IU/ml penicillin/streptomycin, and 0.1 mM non-essential amino acids. The two cell lines were maintained in a humidified 37°C incubator containing 5% carbon dioxide.

### Cell survival rate assay

The survival rates of the NRK-52E and hMSCs were determined using MTT assay kits, as described in a previous study (26). Briefly, 1,500 cells were cultured in each well of 96-well plates at 37°C. After 24 h, the cells were divided into control and experimental groups and the cell survival rates were examined for 4 days. Each day, 100 *μ*l MTT (0.005 g/ml in PBS) were added to each well, according to the manufacturer’s instructions. After 3 h incubation at 37°C, the absorbance (570 nm) was measured under a multi-well enzyme-linked immunosorbent assay reader (SpectraMax Paradigm Multi-Mode Microplate Reader; Molecular Devices, Sunnyvale, CA, USA). The cell survival rate was determined using the following formula: A_570_ experimental group / A_570_ control group × 100%.

### Observation of nuclear condensation

The examine the presence of apoptotic cells exhibiting nuclear condensation, a Hoechst 33342 staining method was used (26,49). The cells (approximately 10^4^) in the control group and experimental group were treated with 10 *μ*g/ml Hoechst 33342 for 5 min. Nuclear condensation was observed under an Olympus BX61 fluorescent microscope (excitation, 352 nm; emission, 450 nm; Olympus Corporation, Tokyo, Japan).

### Sodium dodecyl sulfate (SDS) electrophoresis and western blot analysis

SDS electrophoresis and western blot analysis were performed, according to previous described methods (50,51). Briefly, the cells (approximately 10^7^) were treated with radioimmunoprecipitation assay lysis buffer (50 mM Tris-HCl, 120 mM NaCl, 1 mM EDTA, 1% NP-40, pH 7.5) and centrifuged (16,000 × g) for 10 min at 4°C. The protein was collected from the supernatant layer and the concentration was determined using a BSA Protein Assay Reagent kit (Pierce, Rockford, IL, USA) with a DU-530 spectrophotometer (OD562 nm; Beckman Coulter, Inc., Brea, CA, USA). Equal quantities of protein (60 *μ*g) were separated on a SDS-polyacrylamide gel (13.3%) using GHE320 Mini-STD Vertical Gel Electrophoresis Tank and transferred onto a polyvinylidene difluoride membrane (Millipore, Billerica, MA, USA). The membranes were blocked with 5% milk for 2 h at 25°C and then washed with phosphate-buffered saline (PBS). The membranes were incubated with 5% milk containing the primary antibodies (1:500) for 2 h at 25°C. The membranes were then washed with PBS buffer and treated with secondary antibodies (1:2,000) for 1 h at 25°C. Finally, the proteins were detected using 400 *μ*l Western Lightning Chemiluminescence Reagent Plus (PerkinElmer, Inc., Waltham, MA, USA).

### Determination of oxygen (O_2_^−^) and H_2_O_2_ levels

The levels of O_2_^−^ and H_2_O_2_ were examined using a lucigenin-amplified chemiluminescence technique, as previously described (52,53). Briefly, to determine the levels of H_2_O_2_, 200 *μ*l of the sample (containing 8,000 cells) was treated with 0.2 mmol/l luminol solution (100 *μ*l), followed by examination using a chemiluminescence analyzing system (CLA-FS1; Tohoku Electronic Industrial Co., Ltd., Sendai, Miyagi, Japan). Similarly, to determine the levels of O_2_^−^, 200 *μ*l of the sample (containing 8,000 cells) was treated with 0.1 mmol/l of the lucigenin solution (500 *μ*l), followed by examination using the CLA-FS1 system.

### Statistical analysis

Data were calculated from four independent triplicate experiments and are presented as the means ± standard deviation. Statistical differences between 2 groups were analyzed using the Student’s t-test. A P-value <0.05 was considered to indicate a statistically significant difference.

## Results

### APAP decreases the survival of kidney tubular epithelial cells and hMSCs

**Figure 1:**
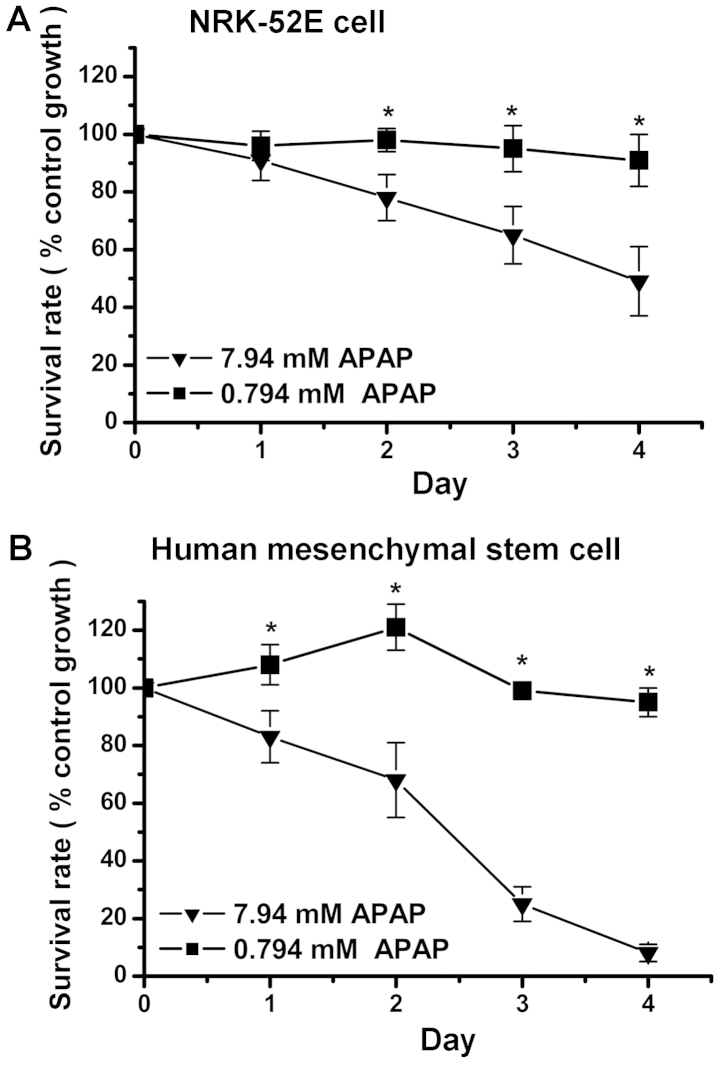
Cell survival rates following treatment with APAP. (A) NRK-52E cells were treated with 7.94 mM (high-dose) and 0.794 mM APAP. (B) hMSCs were treated with 7.94 mM (high-dose) and 0.794 mM APAP. The survival rate was lower in the 7.94 mM APAP-treated hMSCs, compared with the 7.94 mM APAP-treated NRK-52E cells. Data was calculated from four independent experiments and is presented as the means ± standard deviation. The statistical differences between 7.94 mM-treated and 0.794 mM-treated group were analyzed by the Student’s t-test. ^*^P<0.05. APAP, acetaminophen; hMSC, human mesenchymal stem cell.

Previous studies have demonstrated that high-dose APAP (> 5 mM) can decrease the cell survival rate of liver and kidney cells (20–26). Similar to these studies, the present study revealed that high-dose APAP (7.94 mM) reduced cell survival in the NRK-52E kidney tubular epithelial cells (Fig. 1A). Until now, the cytotoxic effects of APAP treatment in human stem cells have not been investigated. The present study is the first, to be best of our knowledge, to demonstrate that high-dose APAP reduced the survival rate of hMSCs (Fig. 1B). The results following low-dose APAP treatment (0.794 mM) revealed no significant cytotoxic effects in the NRK-52E cells or the hMSCs (Fig. 1). The survival rates following high-dose APAP therapy between the NRK-52E cells and hMSCs were also compared. The survival rate on day 3 was ~60% in the APAP-treated NRK-52E cells (Fig. 1A) and ~30% in the APAP-treated hMSCs (Fig. 1B). Therefore, high-dose APAP exerted a more marked cytotoxic effect in the hMSCs, compared with the NRK-52E cells. These findings indicated that APAP induced more damage in the stem cells than in the kidney cells.

### High-dose APAP induces apoptosis and activates the caspase-9/-3 cascade in hMSCs

**Figure 2:**
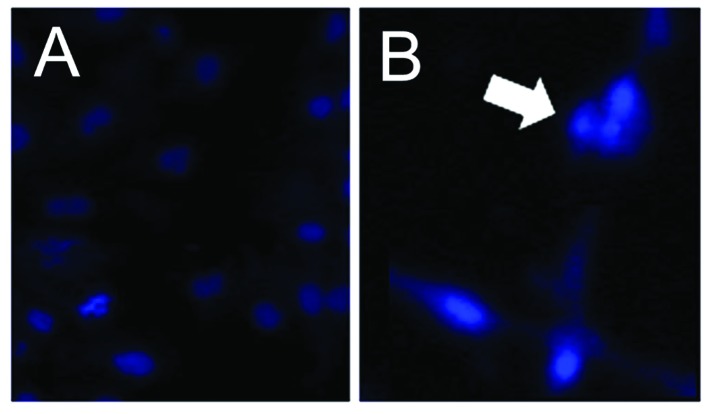
Nuclear condensation. (A) Control group. (B) APAP-treated group. hMSCs were treated with 7.94 mM APAP for 2 days, nuclear condensation (white arrow) was observed in the APAP-treated cells under a phase-contrast microscope at magnifications of x400. APAP, acetaminophen; hMSC, human mesenchymal stem cell.

**Figure 3:**
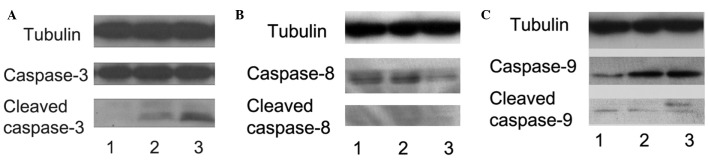
Western blot analysis to determine caspase activation. The activities of (A) caspase-3, (B) caspase-8 and (C) caspase-9 were analyzed on day 3 in the control (lane 1), 0.794 mM APAP-treated (lane 2) and 7.94 mM APAP-treated (lane 3) cells. Ceaved caspase-3 and cleaved caspase-9 were markedly increased in the 7.94 mM APAP-treated cells. APAP, acetaminophen; hMSC, human mesenchymal stem cell.

The present study subsequently examined whether the apoptotic death pathway is involved in hMSC death following high-dose APAP treatment. Upon examination of the nuclear morphology, nuclear condensation, an apoptotic feature (26,54), was identified in the APAP-treated hMSCs (Fig. 2). Thus, the results demonstrated that high-dose APAP induced apoptosis in the hMSCs. Caspase activation triggers apoptosis (49,55). Two major caspase signaling pathways are associated with the apoptosis-caspase-9/-3 and caspase-8/-3 cascades (26,56). Cleaved caspase-3, -8 and -9 were observed following 3 days of APAP treatment using western blot analysis. As shown in Fig. 3, the levels of cleaved caspase-3 and -9 were increased in the high-dose APAP-treated hMSCs (Fig. 3A, lane 3 and Fig. 3C, lane 3); however, the levels of cleaved caspase-8 were unchanged (Fig. 3B). Therefore, these results suggested that high-dose APAP stimulated apoptosis in the hMSCs via the caspase-9/-3 signaling pathway.

### APAP induces the phosphorylation of JNK and p38 in hMSCs

**Figure 4:**
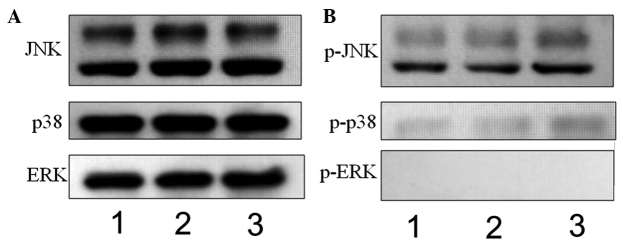
Western blot analysis to determine the expression of mitogen-activated protein kinases, (JNK, p38 and ERK) and their phosphorylation. (A) JNK, p38 and ERK, and (B) p-JNK, p-p38 and p-ERK were observed at 30 min in the control (lane 1), 0.794 mM APAP-treated (lane 2) and 7.94 mM APAP-treated (lane 3) cells. The levels of p-JNK and p-p38 were increased in the 7.94 mM APAP-treated cells. JNK, c-Jun N-terminal kinase; ERK, extracellular signal-regulated kinase; p-, phosphorylated; APAP, acetaminophen.

APAP can induce liver injury via the MAPK signaling pathways (57,58). In the present study, whether APAP also activates the MAPK signaling pathways in hMSCs was examined. JNK, p38 and ERK belong to the MAPK family (59,60). Therefore, the phosphorylation levels of JNK, p38 and ERK were examined using western blot analysis in the present study. As shown in Fig. 4, the levels of p- JNK and p-p38 were increased in the high-dose APAP-treated cells (Fig. 4B, lane 3), compared with the control group (Fig. 4B, lane 1). However, ERK phosphorylation was not observed in the APAP-treated cells (Fig. 4B). These experimental results suggested that APAP activated the JNK/p38 MAPK signaling pathways, but not the ERK MAPK signaling pathway, in the hMSCs.

### APAP stimulates increases in H_2_O_2_ levels in hMSCs

**Figure 5:**
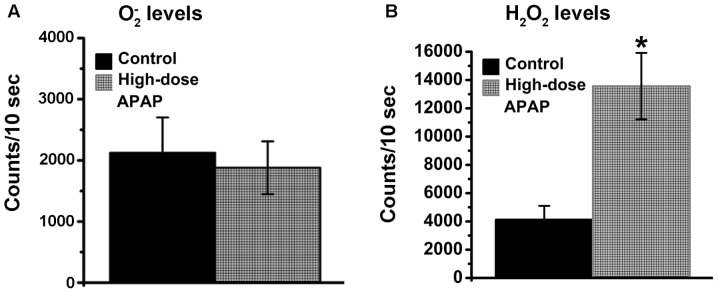
O_2_^−^ and H_2_O_2_ levels. (A) O_2_^−^ levels were determined in the control and high-dose APAP-treated cells. (B) H_2_O_2_ levels were determined in the control and high-dose APAP-treated cells. The levels of O_2_^−^ and H_2_O_2_ levels were measured following 1 h treatment. Data were analyzed from four independent experiments and are presented as the means ± standard deviation. ^*^P<0.05, compared to the control group. O_2_^−^, oxygen; H_2_O_2_ hydrogen peroxide; APAP, acetaminophen.

Previous studies have demonstrated that APAP can induce increases in ROS levels (61,62). In addition, a previous study reported that augmentations in H_2_O_2_ levels are found in APAP-treated kidney cells (26). O_2_^−^ and H_2_O_2_ belong to the ROS family and are normally present in living cells, therefore, the levels of O_2_^−^ and H_2_O_2_ were examined in the present study. As shown in Fig. 5, the O_2_^−^ levels remained constant (Fig. 5A), whereas increases in H_2_O_2_ were found in the APAP-treated hMSCs (Fig. 5B), whereas. Therefore, the APAP-induced augmentation of ROS was associated with H_2_O_2_, but not O_2_^−^, in the hMSCs.

### Vitamin C reduces APAP-induced increases in H_2_O_2_ levels, but does not inhibit APAP-induced cytotoxicity in hMSCs

**Figure 6:**
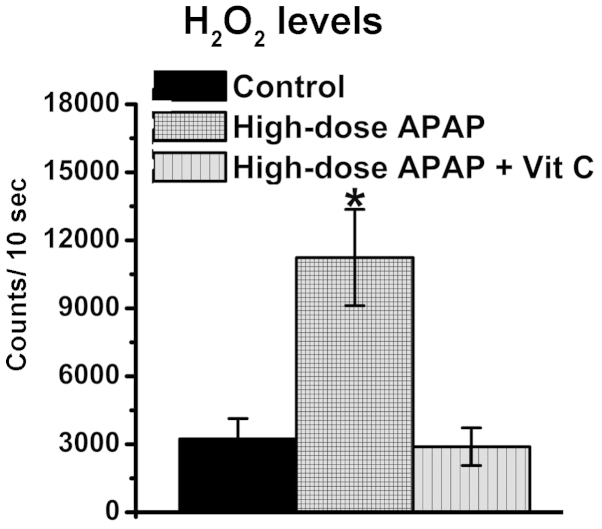
H_2_O_2_ levels. The levels of H_2_O_2_ levels were determined in the control, high-dose APAP-treated and high-dose APAP + 0.5 mM vit C-treated cells. The levels of H_2_O_2_ were measured after 1 h treatment. Data were analyzed from four independent experiments and are presented as the means ± standard deviation.^*^P<0.05, compared to the control group. H_2_O_2_ hydrogen peroxide; APAP, acetaminophen; vit C, vitamin C.

**Figure 7:**
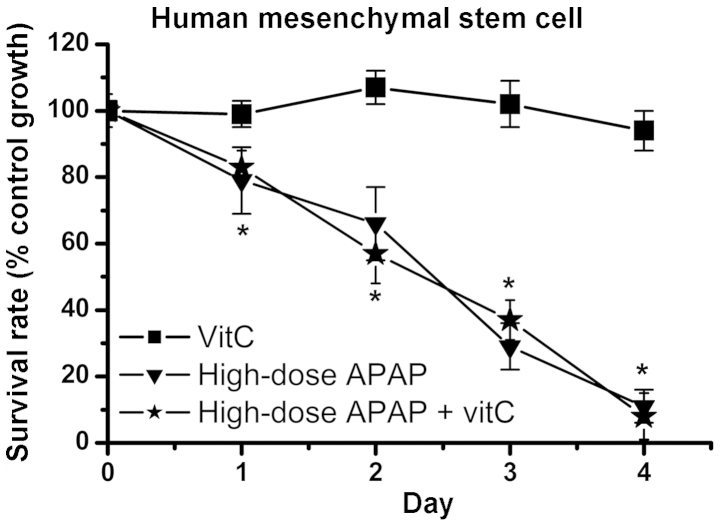
Cell survival rates. The hMSCs were treated with 0.5 mM vit C, high-dose APAP, and high-dose APAP +0.5 mM vit C-treated cells. No significant difference in survival rates were observed between the APAP-treated and APAP + vit C-treated cells. Data were calculated from four independent experiments and are presented as the means ± standard deviation.^*^P<0.05, compared to vitamin C alone group; both APAP group and APAP plus vitamin C group have significant difference. hMSC, human mesenchymal stem cell; APAP, acetaminophen; vit C, vitamin C.

APAP can stimulate elevations in ROS levels causing cellular oxidative stress, which results in hepatotoxicity and nephrotoxicity (16,63). Therefore, several antioxidant drugs that prevent APAP-induced cellular damage have been investigated (64–68). Vitamin C, an antioxidant, was used to inhibit the cytotoxic effects of APAP in the hMSCs in the present study. The resulting data revealed that vitamin C effectively reduced the increases in H_2_O_2_ levels (Fig. 6). Therefore, vitamin C had an antioxidative function in decreasing cellular oxidative stress. Subsequently, whether vitamin C inhibited APAP-induced cytotoxicity in the hMSCs was determined. As shown in Fig. 7, cell survival rates were markedly decreased in the high-dose APAP-treated group and high-dose APAP + vitamin C-treated group, compared to the control group. These findings indicated that inhibition of the increases in H_2_O_2_ did not prevent APAP-induced cytotoxicity in the hMSCs.

The present study was the first, to the best of our knowledge, to demonstrate that high-dose APAP reduced the survival rate of hMSCs, induced the JNK/p38 MAPK signaling pathways and activated the caspase-9/-3 apoptotic death pathway. In addition, the inhibition of increases in the levels of H_2_O_2_ did not rescue the cell survival rate following APAP treatment.

## Discussion

APAP is regarded a safe medicine applied widely to treat pain and fever in clinical cases (69–71). However, high-dose APAP can cause clinical hepatotoxicity and nephrotoxicity (5–7). Previous studies have demonstrated that APAP has antitumor effects in various types of cancer, including liver cancer, breast cancer and neuroblastoma (26,39,43). These studies indicated that APAP can induce cytotoxicity in liver, renal and tumor cells. Therefore, the majority of studies investigating APAP-induced cytotoxic mechanisms have focused on renal, liver and tumor cells (26,39,43,58,62,72,73). The present study was the first, to the best of our knowledge, to demonstrate that APAP also induces cytotoxicity in hMSCs, suggesting APAP not only triggers clinical hepatotoxicity and nephrotoxicity, but it is also harmful to stem cells. Notably, as shown in Fig. 1, the present study demonstrated that APAP exerts a more marked cytotoxic effect in hMSCs than in renal tubular cells. Stem cells are important in fetal development, and stem cell dysfunction may be harmful to fetus growth. In addition, previous studies have demonstrated that APAP can cross the placenta (44,45). Therefore, the results of the present study suggested the requirement for caution when treating pregnant females with APAP for pain and fever.

The activation of apoptosis and necrosis have been found in liver and renal cells following APAP treatment in different animal and cell models (31,32). The majority of studies have reported that APAP-induced apoptotic death in liver and renal cells is associated with caspase-3 activation (74–76). There are two major caspase cascades, caspase-9/-3 and caspase-8/-3 cascades (26,54,55). The caspase-9/-3 cascade is linked to mitochondrial dysfunction and the caspase-8/-3 casecade is associated with death receptor signal transduction. APAP-induced liver and renal injury has been observed to trigger the caspase-9/-3 pathway (11,77). In addition, activation of the caspase-9/-3 cascade is also found in APAP-treated hepatoma cells (26). In the present study, the data revealed that APAP activated caspase-9 and -3 signaling in the hMSCs but did not activate caspase-8 (Fig. 3). Taken together, these studies indicated that mitochondrial damage is an important factor that results in cell death in renal cells, liver cells, hepatoma cells and stem cells following APAP treatment.

The MAPK signaling pathways undergo three major phosphorylation reactions: ERK, JNK and p38 phosphorylation (59,60). Previous studies have demonstrated that APAP can induce acute liver injury via the JNK and ERK phosphorylation signaling pathways (57,58). A previous study found that APAP-induced liver damage not only activates JNK and ERK phosphorylation, but also induces p38 phosphorylation in mouse models (78), although the common signaling pathways are the ERK and JNK phosphorylation pathways. In the present study, JNK and p38 phosphorylation were observed in the APAP-treated hMSCs, however, ERK phosphorylation was not observed (Fig. 4). The observation of ERK phosphorylation in the APAP-treated liver cells, but not in the stem cells remains to be elucidated and requires investigation in the future.

Previous studies have demonstrated that high-dose APAP-induced hepatotoxicity and nephrotoxicity are associated with increases in ROS levels (25,79–81). Severalantioxidants against APAP-induced cytotoxicity have been investigated, including green tea, honey, tofu and NAC (13,80,82–85). O_2_^−^ and H_2_O_2_ belong to the ROS family and are produced by the electron transport chain. O_2_^−^ can be removed by superoxide dismutase, and H_2_O_2_ can be removed by glutathione system (19,26,85). NAC, a precursor for glutathione synthesis, can effectively reduce H_2_O_2_ levels and has been applied as a treatment method for APAP-induced hepatotoxicity and nephrotoxicity in clinical cases (17–19). The levels of O_2_^−^ and H_2_O_2_ can be determined in APAP-treated stem cells. The present study demonstrated that APAP stimulated increases in the levels of H_2_O_2_, but not O_2_^−^, in human stem cells. This result is similar to a previous study, in which only increases in H_2_O_2_ levels were found in APAP-treated Hep3B cells (26). In addition, the present study further demonstrated that vitamin C effectively reduced APAP-induced elevations in H_2_O_2_, but does not inhibit APAP-induced cytotoxicity, in human cells. This result indicated that there are unknown cellular effects, in addition to augmentations in the levels of ROS, resulting in APAP-induced cytotoxicity in human stem cells. The present study demonstrated that antioxidants agents prevented APAP-induced damage in liver and renal cells, but not in stem cells.

In conclusion, this study was the first, to the best of our knowledge, to demonstrate that APAP induced the p38/JNK MAPK signaling pathway, activated the caspase-9/-3 cascade and decreased survival rate in human stem cells. The present study also revealed that APAP-induced cytotoxic effects were more marked in stem cells than in renal cells, and antioxidants did not prevent stem cell damage following APAP treatment.
